# Harnessing Phytochemicals and Nanotechnology Synergy for Molecular, Epigenetic, and Microbiota-Driven Regulation in Type 2 Diabetes Mellitus

**DOI:** 10.3390/pharmaceutics18010113

**Published:** 2026-01-15

**Authors:** Gagan Prakash, Anis Ahmad Chaudhary, Ruchita Tanu, Mohamed A. M. Ali, Fehmi Boufahja, Pushpender K. Sharma, Sudarshan Singh Lakhawat, Tejpal Yadav, Navneet Kumar Upadhyay, Vikram Kumar

**Affiliations:** 1 Amity Institute of Biotechnology, Amity University Rajasthan, Jaipur 303002, Rajasthan, India; 2 Department of Biology, College of Science, Imam Mohammad Ibn Saud Islamic University (IMSIU), Riyadh 11564, Saudi Arabia; 3 Amity Institute of Pharmacy, Amity University Rajasthan, Jaipur 303002, Rajasthan, India

**Keywords:** type 2 diabetes mellitus, phytochemicals, nanocarriers, molecular pathways, gut microbiota, precision medicine

## Abstract

Type 2 diabetes mellitus (T2DM) is a multifaceted metabolic disorder marked by impaired insulin action, pancreatic β-cell dysfunction, and the involvement of several interconnected mechanisms, including inflammation, oxidative stress, and epigenetic alterations. Despite progress in conventional therapies, achieving durable glycemic control and minimizing complications remain major challenges. This review discusses the emerging role of bioactive phytochemicals—such as curcumin, berberine, resveratrol, flavonoids, and polysaccharides—in modulating essential molecular pathways including AMPK, PI3K/AKT, and cAMP/PKA, which contribute to enhanced insulin sensitivity, glucose regulation, and β-cell protection. These natural compounds also influence gut microbiota modulation and epigenetic mechanisms, offering additional metabolic and anti-inflammatory benefits. This review synthesizes evidence from peer-reviewed studies published between 2000 and 2024, incorporating bibliometric trends showing an increasing research focus on phytochemicals for T2DM management. However, limitations such as low solubility, instability, and poor absorption restrict their clinical application. Advances in nanotechnology-based delivery systems, including nanoparticles, liposomes, and nanoemulsions, have shown potential to overcome these barriers by improving stability, bioavailability, and targeted delivery of phytochemicals. The integration of gut microbiota modulation with nanocarrier-enabled phytochemical therapy supports a precision medicine approach for managing T2DM. Preliminary clinical evidence highlights significant improvements in glycemic control and inflammatory status, yet further large-scale, well-controlled trials are essential to ensure safety, optimize dosages, and standardize combination regimens. Overall, phytochemical therapies, reinforced by nanotechnology and microbiota modulation, present a promising, safe, and holistic strategy for T2DM management. Continued interdisciplinary research and clinical validation are crucial for translating these advances into effective therapeutic applications and reducing the global diabetes burden.

## 1. Introduction

Type 2 diabetes mellitus (T2DM) is a chronic metabolic disorder characterized by persistent hyperglycemia due to a combination of insulin resistance and relative insulin deficiency, driven by progressive β-cell dysfunction and impaired glucose regulation [1,2,3]. Unlike type 1 diabetes, T2DM develops gradually, predominantly affecting adults but increasingly seen in younger populations, paralleling rising obesity rates worldwide [4,5]. The global burden of T2DM continues to escalate, with over 529 million affected individuals currently and projections nearing 1.3 billion by 2050, especially in low- and middle-income regions undergoing rapid urbanization and lifestyle transitions [6,7]. This growing epidemic contributes substantially to disability, premature mortality, and healthcare costs, highlighting the urgent need for improved prevention and management strategies [6,8,9].

Despite advances in pharmacological treatments, managing T2DM remains challenging due to lifelong medication requirements, side effects, and incomplete targeting of the disease’s multifaceted ethology [10,11]. Many conventional therapies do not adequately address lifestyle and dietary influences. Consequently, there is growing scientific interest in complementary approaches, including dietary modifications and the use of natural products and herbal remedies, which may improve glycemic control and mitigate disease progression [12,13,14]. However, the therapeutic potential of these compounds is often limited by poor bioavailability and stability. Nanotechnology-based delivery systems—such as liposomes, nano emulsions, and polymeric nanoparticles—offer promising solutions to enhance the absorption, stability, and targeted delivery of bioactive natural compounds like curcumin, berberine, and resveratrol, potentially translating laboratory successes into effective clinical outcomes [6].

Nanotechnology-enabled delivery platforms represent a novel and synergistic strategy to maximize the efficacy of natural products in T2DM management. These innovations support the growing paradigm of personalized and precision nutrition tailored to individual metabolic profiles and disease risk [15,16]. Given the global burden and complex ethology of T2DM, a comprehensive evaluation of natural product-based therapies—alongside lifestyle and technological advances—is critical for developing safer, more effective, and multifactorial treatment strategies to curb this epidemic and improve patient outcomes worldwide [17,18,19].

Combining nanotechnology with phytochemical-based treatments offers a promising and innovative approach to managing diabetes, merging traditional remedies with cutting-edge science. This integration improves the accuracy and effectiveness of therapies while promoting sustainable, personalized care strategies to address the growing global challenge of type 2 diabetes mellitus.

This review aims to systematically assess the antidiabetic potential of key phytochemicals, elucidate their structure–activity relationships (SAR), and consolidate current findings on nanocarrier-based delivery systems that improve their stability, bioavailability, and therapeutic effectiveness in type 2 diabetes mellitus (T2DM). Additionally, it emphasizes important natural sources of bioactive compounds, evaluates quantitative efficacy data, and identifies existing research gaps to inform future clinical applications. A bibliometric trend analysis (2000–2024) shows a rising number of studies investigating phytochemicals and T2DM, which supports the relevance of this review.

## 2. Type 2 Diabetes Mellitus

T2DM primarily results from insulin resistance in peripheral tissues including muscle, liver, and adipose tissue, where cells inadequately respond to insulin leading to reduced glucose uptake and persistent hyperglycemia. This resistance is exacerbated by inflammatory cytokines, elevated free fatty acids, and lipid accumulation that impair insulin receptor substrate activation and downstream signalling pathways, culminating in increased hepatic glucose production [1,20]. Concurrently, pancreatic β-cell dysfunction occurs due to oxidative stress, mitochondrial and endoplasmic reticulum stress, and accumulation of reactive oxygen species, leading to β-cell apoptosis and diminished insulin secretion. Although β-cell hyperplasia initially compensates, sustained metabolic stress eventually impairs secretory capacity, advancing disease progression [1,21]. This interplay creates a vicious cycle whereby worsening β-cell failure drives increased insulin resistance and chronic hyperglycemia, which induces further cellular damage through oxidative stress, inflammation, and the formation of advanced glycation end-products that contribute to vascular and neuronal complications [1]. Genetic factors, such as polymorphisms in TCF7L2 and PPARG, alongside epigenetic influences including obesity-related DNA methylation changes, modify individual susceptibility and disease trajectory by affecting β-cell function and insulin action [22,23]. Additionally, dysregulated α-cell secretion of glucagon exacerbates hyperglycemia by increasing hepatic glucose production, while impaired paracrine interactions between α- and β-cells further destabilize glucose homeostasis [24]. This multifactorial pathophysiology underscores the complex molecular and cellular mechanisms driving T2DM [25,26].

## 3. Insulin Secretion and Resistance

### 3.1. Insulin Resistance

Insulin resistance is a pathological condition in which the body’s cells, primarily in skeletal muscle, liver, and adipose tissue, exhibit diminished responsiveness to normal insulin levels, impairing glucose uptake and utilization for energy. This state disrupts the ability of insulin to suppress hepatic glucose production and reduce blood glucose effectively, despite elevated insulin levels [27,28]. To compensate, the pancreas initially increases insulin secretion, resulting in hyperinsulinemia; however, chronic metabolic stress eventually leads to β-cell exhaustion, failure of compensation, and persistent hyperglycemia, culminating in T2DM [21]. Insulin resistance is closely associated with obesity, chronic inflammation, and excess caloric intake, which further induce metabolic abnormalities such as dyslipidemia, hypertension, and systemic inflammation, thereby escalating the risk of cardiovascular disease and non-alcoholic fatty liver disease [27]. This condition can develop 10 to 15 years prior to overt T2DM and is measurable through precise methods like the hyperinsulinemic-euglycemic clamp or surrogate indices such as HOMA-IR. Early detection and interventions aimed at reducing insulin resistance are critical to preventing or delaying the onset of diabetes and related complications [27,29,30].

### 3.2. Beta-Cell Dysfunction

Beta-cell dysfunction refers to the inability of pancreatic beta cells, located in the islets of Langerhans, to produce and secrete sufficient insulin in response to rising blood glucose. Healthy beta cells are essential for promptly adjusting insulin secretion according to blood glucose fluctuations, ensuring glucose homeostasis [31,32].

The progressive nature of beta-cell dysfunction is a hallmark of T2DM. Chronic exposure to high levels of glucose (glucotoxicity) and fatty acids (lipotoxicity), mediated by mechanisms such as inflammation, oxidative stress, mitochondrial dysfunction, and endoplasmic reticulum stress, all contribute to beta-cell injury and apoptosis [2,33,34]. Additional contributors include genetic susceptibility, ageing, and amyloid accumulation within islets.

Beta-cell dysfunction is a central factor in the transition from prediabetes to overt T2DM. By the time T2DM is clinically diagnosed, beta-cell loss is often advanced and irreversible, highlighting the need for early detection and targeted intervention. Preserving beta-cell function remains a major therapeutic goal, as ongoing decline leads to worsening glycemic control and elevated risk of diabetes-related complication [35,36,37].

## 4. Signalling Pathway and Therapeutic Interventions

Two critical intracellular signalling pathways often disrupted in T2DM are the AMP-activated protein kinase (AMPK) and phosphoinositide 3-kinase/protein kinase B (PI3K/AKT) pathways. AMPK acts as an essential cellular energy sensor that promotes glucose uptake, enhances fatty acid oxidation, and inhibits gluconeogenesis and lipid synthesis, thereby improving insulin sensitivity and lowering blood glucose levels. Pharmacological drugs like metformin and thiazolidinediones exert part of their antidiabetic effects by activating AMPK. The PI3K/AKT pathway plays a pivotal role in mediating insulin signalling by regulating glucose transporter 4 (GLUT4) translocation to the plasma membrane, glycogen synthesis, and cell survival. Dysregulation of PI3K/AKT signalling causes impaired glucose homeostasis and insulin resistance, hallmark features of T2DM. Natural compounds such as berberine, quercetin, and resveratrol have been shown to activate AMPK and enhance insulin signalling, partly through supporting PI3K/AKT activity, reducing inflammation, and decreasing oxidative stress. Some natural agents also affect the cyclic AMP/protein kinase A (cAMP/PKA) pathway, offering additional metabolic regulation benefits. Collectively, targeting AMPK, PI3K/AKT, and cAMP/PKA pathways through both pharmacological and natural therapies represents a comprehensive approach to improving insulin action and managing diabetes [38,39].

### 4.1. PI3K/AKT Pathway

**Figure 1:**
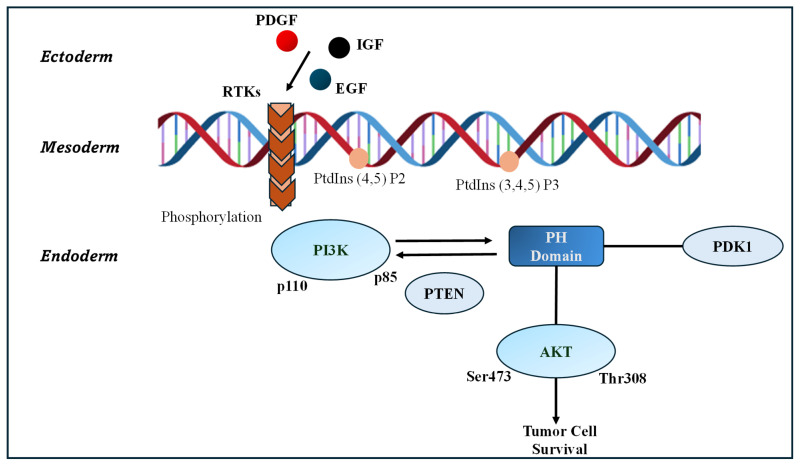
**Growth Factor-Mediated PI3K/AKT Pathway Activation:** Growth factors (PDGF, IGF, EGF) activate receptor tyrosine kinases (RTKs), leading to phosphorylation and conversion of PtdIns (4,5) P2 to PtdIns (3,4,5) P3 by PI3K. PTEN negatively regulates this step. PtdIns (3,4,5) P3 recruits PDK1 and AKT to the membrane, enabling AKT phosphorylation at Thr308 and Ser473. Activated AKT drives tumour cell survival and regulates growth, metabolism, and proliferation.

Figure 1 illustrates the PI3K/AKT signalling pathway, a critical molecular cascade that regulates cellular development, metabolism, survival, and proliferation. The activation process initiates at the plasma membrane when growth factors such as platelet-derived growth factor (PDGF), insulin-like growth factor (IGF), and epidermal growth factor (EGF) bind to receptor tyrosine kinases (RTKs), inducing receptor dimerization and autophosphorylation. This activation recruit’s phosphoinositide 3-kinase (PI3K), composed of catalytic (p110) and regulatory (p85) subunits, which phosphorylates phosphatidylinositol 4,5-bisphosphate (PIP2) to generate phosphatidylinositol 3,4,5-trisphosphate (PIP3). The pathway intensity is finely regulated by phosphatase and tensin homologue (PTEN), which dephosphorylates PIP3 back to PIP2, thus negatively controlling signal propagation [40,41,42].

PIP3 serves as a docking platform for pleckstrin homology (PH) domain–containing proteins such as phosphoinositide-dependent kinase 1 (PDK1) and protein kinase B (AKT). PDK1 phosphorylates AKT at threonine 308, while subsequent phosphorylation at serine 473 by other kinases results in full AKT activation. Activated AKT then modulates diverse downstream effectors responsible for gene transcription, protein synthesis, glucose metabolism, and cell survival. In insulin-responsive tissues—including skeletal muscle, liver, and adipose tissue—impaired AKT activation disrupts glucose uptake and promotes hepatic glucose overproduction, which are key contributors to insulin resistance and hyperglycemia in T2DM. Dysregulation of PI3K/AKT signalling is therefore central to the pathophysiology of T2DM and presents a valuable therapeutic target for restoring metabolic homeostasis [38,43].

### 4.2. MAPK Pathway

**Figure 2:**
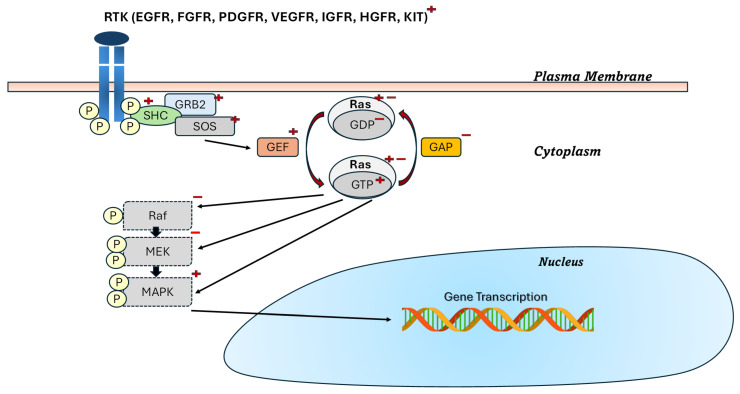
**RTK-Mediated MAPK/ERK Signalling Pathway:** Binding of ligands to RTKs such as EGFR, FGFR, PDGFR, VEGFR, IGFR, HGFR, and KIT induces receptor autophosphorylation. This phosphorylation recruit’s adaptor proteins SHC, GRB2, and SOS, which facilitate the activation of Ras through GDP to GTP exchange. Active Ras initiates the Raf–MEK–MAPK kinase cascade, resulting in the phosphorylation and activation of MAPK. The activated MAPK translocate into the nucleus, where it regulates gene transcription and modulates cellular processes.

The Mitogen-Activated Protein Kinase (MAPK) signalling cascade is a pivotal intracellular pathway regulating cellular growth, differentiation, and gene expression by transmitting extracellular signals from RTKs to the nucleus. Activation of this pathway initiates when growth factors bind to RTKs such as EGFR, FGFR, PDGFR, VEGFR, IGFR, HGFR, and KIT, triggering receptor autophosphorylation at multiple tyrosine residues that serve as docking sites for adaptor proteins SHC and GRB2. These adaptors recruit the guanine nucleotide exchange factor SOS, which catalyzes the exchange of GDP for GTP on Ras, converting it to its active form. Ras-GTP, carefully regulated by GTPase-activating proteins (GAPs) that accelerate GTP hydrolysis to GDP, triggers a phosphorylation cascade involving the sequential activation of Raf, MEK, and MAPK. Activated MAPK translocate to the nucleus, where it modulates transcription factors to alter gene expression, thus influencing cellular proliferation and differentiation. This tightly controlled cascade depicted in Figure 2 represents a fundamental mechanism by which cells respond to external stimuli, with significant implications in metabolic regulation and disease pathogenesis, including type 2 diabetes mellitus [44,45,46].

### 4.3. cAMP/PKa Pathway

**Figure 3:**
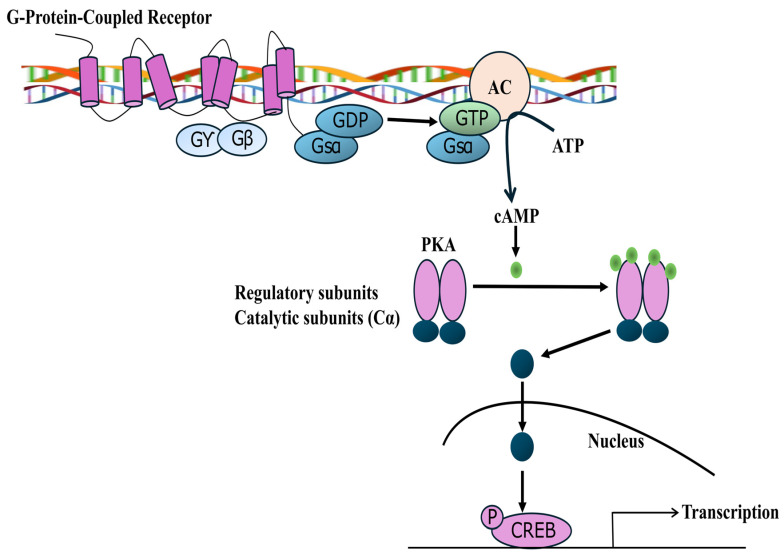
**cAMP/PKA Signalling Pathway: From GPCR Activation to CREB-Mediated Transcription;** succinctly illustrates the molecular sequence by which extracellular signals are transduced into nuclear gene regulation. The process initiates with ligand binding to a GPCR on the cell membrane, triggering a conformational shift that activates the heterotrimeric G protein via GDP-GTP exchange on the Gα subunit (Gsa). The active GTP-bound Gα dissociates from Gβγ and stimulates adenylyl cyclase, converting ATP to the second messenger cAMP. Elevated cAMP then activates PKA by releasing catalytic subunits (Cα), which enter the nucleus to phosphorylate CREB. Phosphorylated CREB binds DNA at cAMP response elements to regulate transcription of genes central to cellular survival, growth, and metabolism.

The cAMP/PKA signalling pathway, as demonstrated in Figure 3, is a critical intracellular mechanism by which extracellular signals are converted into cellular responses. Activation begins when a ligand binds to a G-protein-coupled receptor (GPCR) on the cell membrane, inducing conformational changes that facilitate the exchange of GDP for GTP on the Gα subunit (Gsa). The activated GTP-bound Gα subunit subsequently interacts with adenylyl cyclase (AC), catalyzing the conversion of ATP to the second messenger cyclic AMP (cAMP). Elevated cAMP levels lead to the activation of protein kinase A (PKA) by releasing its catalytic subunits (Cα) from regulatory subunits. These catalytic subunits translocate to the nucleus where they phosphorylate transcription factors such as CREB, thus regulating gene transcription critical for processes including cell growth, metabolism, and survival [47,48,49].

Recent studies have further elucidated the spatial and temporal regulation of this pathway, highlighting the roles of compartmentalized signalling and PKA isoform-specific functions that enable precise cellular outcomes. Notably, emerging research demonstrates the pathway’s involvement in metabolic regulation and diseases such as diabetes, with interventions targeting cAMP/PKA signalling showing promise in restoring impaired insulin secretion and glucose homeostasis. For instance, myricetin has been reported to enhance glucose-stimulated insulin secretion via the cAMP-PKA pathway, emphasizing the therapeutic potential of modulating this cascade [47,50].

## 5. Important Traditional Medicinal Herbs for Diabetes

Natural phytochemicals—bioactive compounds found in medicinal herbs—have been deeply embedded in traditional medicinal systems worldwide, notably Ayurveda and herbal medicine, for the management and prevention of diabetes mellitus. Classic references cite herbs such as fenugreek (*Trigonella foenum-graecum* L.), bitter melon (*Momordica charantia* L.), cinnamon (*Cinnamomum verum* J.Presi), turmeric (*Curcuma longa*), and gymnema (*Gymnema sylvestre* R.Br.) for their antidiabetic properties in ancient scripts and household remedies [51,52,53,54,55,56,57,58,59]. These plants contain diverse phytochemicals like flavonoids, alkaloids, terpenoids, and saponins, which have demonstrated significant blood glucose-lowering and insulin-sensitizing effects in modern preclinical and clinical studies. For instance, flavonoids and phenolic acids reduce oxidative stress and inflammation, both central to diabetes pathogenesis, while gymnemic acids from Gymnema sylvestre suppress sugar absorption and enhance insulin action. Evidence also reveals that fenugreek and bitter melon possess compounds capable of improving glycemic control and modulating glucose metabolism in people with type 2 diabetes [12,60,61,62].

Integration of natural phytochemicals through herbal and Ayurvedic therapies offers holistic benefits for managing type 2 diabetes mellitus. Bitter gourd (*Momordica charantia*), neem (*Azadirachta indica*), and turmeric are routinely used in decoctions and supplements to detoxify blood, regulate blood sugar, and alleviate insulin resistance [57,63,64,65]. Ayurvedic protocols prescribe “sugar destroyer” herbs like Gudmar (*Gymnema sylvestre*), fenugreek, and triphala, supporting not only lower glucose absorption but balanced metabolism and digestion [51,52,53,59,66,67]. Recent trials show that plant extracts such as those from Artemisia afra and Persea americana (avocado) can significantly reduce fasting blood glucose, by activating molecular pathways like PKB/Akt, thus improving glucose uptake in tissues. Additionally, substances like berberine match the efficacy of conventional medications such as metformin for glucose and lipid regulation. Modern science validates these ancient remedies, as a multitude of phytochemicals—from flavonoids to glycosides—target inflammation, oxidative stress, and insulin sensitivity to support diabetes management [61,62,68,69,70].

Recent research profoundly underscores the significance of natural phytochemicals and herbal therapies in type 2 diabetes, not only for symptom control but potential disease modification. These botanical interventions, rich in antioxidants and anti-inflammatory compounds, improve insulin sensitivity, reduce complications, and are increasingly accepted as safe, cost-effective complements to conventional treatment. Their use is corroborated by evidence both from traditional knowledge and contemporary pharmacological studies, with current references pointing toward a robust future for integrative diabetes care harnessing the power of medicinal herbs and phytochemicals [60,61,62,70].

## 6. DM-2 Regulating Mechanism of Natural Phytochemicals

### 6.1. Insulin Secretion and Sensitivity

Phytochemicals derived from dietary and medicinal plants play a significant role in improving insulin sensitivity and secretion, thereby supporting glucose homeostasis disrupted in T2DM. These natural compounds engage multiple mechanisms including activation of key molecular pathways such as PI3K/Akt and AMPK, which enhance glucose uptake in peripheral tissues, stimulate pancreatic β-cell function to increase insulin release, and mitigate oxidative stress and inflammation—two major contributors to insulin resistance. Additionally, certain phytochemicals modulate gene expression and microRNAs to promote β-cell survival and overall metabolic regulation. Key examples include polyphenols like quercetin and resveratrol that activate PI3K/Akt and AMPK signalling, saponins such as ginsenosides that facilitate GLUT4 translocation, and alkaloids like berberine known for stimulating insulin secretion and suppressing hepatic gluconeogenesis. Flavonoids including kaempferol and naringenin protect β-cells and enhance insulin release, while antioxidants such as catechins and curcumin further improve insulin signalling by reducing oxidative damage. These phytochemicals, often used in nanoformulations to improve bioavailability, present promising adjunctive therapies for T2DM management by targeting multiple facets of insulin resistance and β-cell dysfunction [71].

### 6.2. Antioxidant and Anti-Inflammatory Effort

In T2DM, oxidative stress and chronic low-grade inflammation constitute a vicious cycle that significantly contributes to pancreatic β-cell dysfunction, impaired insulin secretion, and insulin resistance. Persistent hyperglycemia accelerates the production of reactive oxygen species (ROS), which induce oxidative damage and activate inflammatory pathways, exacerbating metabolic defects [72,73]. The oxidative insult leads to β-cell injury and diminishes insulin sensitivity by disrupting insulin receptor signalling and promoting pro-inflammatory cytokine release such as TNF-α and IL-6. Phytochemicals including curcumin, resveratrol, quercetin, and catechins counter these effects by scavenging ROS, upregulating endogenous antioxidant enzymes (e.g., superoxide dismutase, catalase, glutathione peroxidase), and inhibiting inflammatory mediators, thereby protecting β-cells and improving glucose regulation [74,75]. Advanced nanoformulations of these bioactive enhance their stability and bioavailability, facilitating their therapeutic translation [76].

The accompanying figure illustrates this mechanism, contrasting the pathological sequence of hyperglycemia-driven ROS overproduction and oxidative stress leading to β-cell damage and insulin resistance, against the protective role of phytochemicals. By effectively scavenging ROS, enhancing antioxidant enzyme activity, and suppressing inflammatory cytokines, phytochemicals restore insulin sensitivity and β-cell function. This dual antioxidant and anti-inflammatory action underscore the therapeutic potential of plant-derived compounds as multi-target adjuncts in managing T2DM, offering promising avenues to mitigate disease progression and complications [60,77,78].

### 6.3. Epigenetic Regulation

Epigenetic modifications—including DNA methylation, histone acetylation and deacetylation, and microRNA (miRNA) regulation—are crucial in the development and progression of T2DM by altering gene expression without changing the DNA sequence, thereby contributing to insulin resistance, β-cell dysfunction, and metabolic memory [79,80]. Key mechanisms involve DNA methylation changes such as hypomethylation of inflammatory gene promoters increasing oxidative stress, and hypermethylation of insulin-related genes like PPARG and KCNQ1 leading to impaired insulin secretion and sensitivity. Dysregulated histone modifications, including elevated histone deacetylase 7 (HDAC7) in pancreatic β-cells, suppress insulin output, while imbalances in histone acetyltransferases (HATs) and HDACs further disrupt glucose metabolism. Aberrant microRNA regulation—for instance, overexpression of miR-29a and dysregulation of miR-375—negatively impacts insulin signalling and β-cell function [79,81,82].

The Table 1 include phytochemicals that act as promising epigenetic modulators to improve metabolic outcomes in T2DM. Curcumin targets DNA methylation at the Nrf2 promoter, enhancing antioxidant defences and reducing oxidative damage, with evidence from both preclinical and clinical studies [83,84,85,86]. Flavonoids such as quercetin and kaempferol modulate histone acetylation and microRNAs, improving insulin signalling and protecting β-cells, supported mainly by preclinical data [82,87]. Sulforaphane serves as a histone deacetylase (HDAC) inhibitor, boosting insulin sensitivity and reducing inflammation, as demonstrated in preclinical and early clinical studies [79,88]. Soy isoflavones influence DNA methylation and histone acetylation, resulting in improved glycemic control and lipid metabolism, with clinical evidence backing their efficacy [80,89]. Lignans also regulate DNA methylation to enhance glucose tolerance and lower inflammation according to clinical research [89]. Berberine impacts microRNA regulation (including miR-21 and miR-29), which improves insulin sensitivity and reduces β-cell apoptosis, based on preclinical studies [79,88]. Collectively, these phytochemicals offer multi-faceted epigenetic interventions that are promising for T2DM prevention and therapy, underscoring the potential of natural products as low-toxicity epigenetic drugs.

**Table 1:** Phytochemicals Targeting Epigenetic Mechanisms in T2DM.

Compound/Class	Primary Epigenetic Target(s)	Key Effects	Evidence Level	References
Curcumin	DNA methylation (*Nrf2* promoter)	Enhances antioxidant defence; reduces oxidative damage	Preclinical and clinical	[72,88]
Flavonoids (e.g., Quercetin, Kaempferol)	Histone acetylation, miRNA modulation	Improves insulin signalling and β-cell protection	Preclinical	[80,89]
Sulforaphane	HDAC inhibition	Enhances insulin sensitivity; reduces inflammation	Preclinical and early clinical	[72,79]
Soy Isoflavones	DNA methylation; histone acetylation	Improves glycemic control and lipid metabolism	Clinical	[80,89]
Lignans	DNA methylation	Improves glucose tolerance; reduces inflammation	Clinical	[89]
Berberine	miRNA regulation (e.g., miR-21, miR-29)	Enhances insulin sensitivity; reduces β-cell apoptosis	Preclinical	[79,88]

### 6.4. Regulation of Signalling Pathway

**Figure 4:**
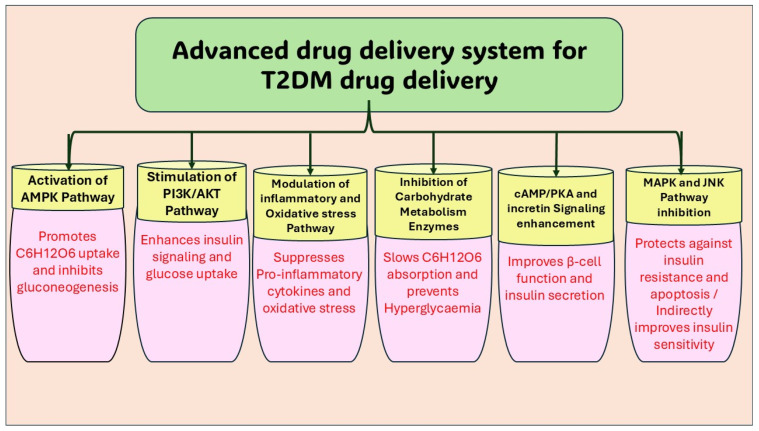
Phytochemicals as Multi-Target Modulators of Signalling Pathways in T2DM Therapy.

The antidiabetic effects of natural phytochemicals largely stem from their ability to modulate key signalling pathways involved in insulin sensitivity, glucose uptake, and metabolic regulation. As illustrated in Figure 4, these compounds exert multi-targeted actions such as activating the PI3K/AKT and AMPK pathways, mitigating inflammatory and oxidative stress pathways, and enhancing insulin receptor sensitivity. Polyphenols and flavonoids, including quercetin and resveratrol, activate PI3K/AKT and AMPK signalling to promote GLUT4 translocation and increase glucose uptake in muscle and adipose tissue. Bioactive phytochemicals such as berberine, resveratrol, and epigallocatechin gallate (EGCG) boost AMPK activation, improving lipid and glucose metabolism, reducing oxidative stress, and suppressing hepatic gluconeogenesis, thus lowering blood glucose levels. Others, like quercetin and curcumin, regulate gene expression and enzymes related to insulin resistance and β-cell protection. Emerging studies also highlight phytochemicals’ modulation of upstream elements, including adaptor proteins and growth factor receptors, enhancing insulin action, while inhibition of negative regulators like protein tyrosine phosphatase 1B (PTP1B) further improves receptor activity. Collectively, these diverse mechanisms highlight the broad therapeutic potential of phytochemicals to counteract oxidative stress, inflammation, and impaired glucose metabolism in T2DM [90,91,92,93].

### 6.5. Modulation of mRNA

Phytochemicals have significant effects on both transcriptional and post-transcriptional regulation in type 2 diabetes mellitus (T2DM), impacting gene expression, mRNA stability, and protein translation. Bioactive compounds such as polyphenols, flavonoids, alkaloids, and terpenoids interact directly with transcription factors or chromatin-modifying enzymes, thereby modulating the expression of genes involved in β-cell function, insulin signalling, and glucose metabolism [88,94,95].

For instance, curcumin activates the Nrf2 pathway by reducing methylation of its promoter, which enhances antioxidant defences and protects β-cells against oxidative damage. Berberine regulates microRNAs such as miR-21 and miR-29, improving insulin sensitivity and reducing β-cell apoptosis [82]. These transcriptional and epigenetic modifications collectively promote better metabolic homeostasis and glycemic control.

### 6.6. Gut Microbiota Modulation

Plant-derived compounds such as polyphenols, flavonoids, curcumin, catechins, and polysaccharides beneficially reshape the gut microbial populations by enriching beneficial bacteria like *Akkermansia*, *Bifidobacterium*, and *Faecalibaculum*, while suppressing harmful species such as *Bilophila* and *Clostridium* [96,97,98]. These shifts promote increased production of microbial metabolites, including short-chain fatty acids (SCFAs), bile acids, and tryptophan derivatives, which improve insulin sensitivity, reduce chronic inflammation, and support the integrity of the gut barrier. Enhanced gut barrier integrity decreases endotoxemia caused by lipopolysaccharide leakage, thereby mitigating systemic inflammation and metabolic dysfunction [97,99,100,101,102,103].

Phytochemicals not only modulate microbial composition but also exert direct anti-inflammatory and antioxidant effects that protect pancreatic β-cells from oxidative stress and inflammatory damage, preserving insulin secretion capacity [77,78,104]. Furthermore, microbial metabolites derived from phytochemical metabolism activate key host pathways such as AMP-activated protein kinase (AMPK) and SIRT1, which regulate glucose uptake, lipid oxidation, and energy homeostasis, thereby ameliorating insulin resistance. Different classes of phytochemicals exert distinct modulatory effects on the gut microbiota and metabolic outcomes: polyphenols enhance SCFA production and improve insulin sensitivity; flavonoids increase microbial diversity and glycemic control; polysaccharides promote glucose metabolism and reduce oxidative stress; curcumin improves β-cell function through anti-inflammatory signalling; and catechins enhance glucose homeostasis and lower endotoxemia [39,105,106,107,108,109,110,111]. These comprehensive interactions position phytochemical-driven gut microbiota modulation as a valuable therapeutic modality for T2DM management.

## 7. Functional Phytochemicals in the Natural Product for Managing of T2DM

A diverse range of phytochemicals derived from natural sources—including flavonoids (quercetin, kaempferol, rutin), polyphenols (resveratrol, curcumin, catechins), alkaloids (berberine, gymnemic acid), terpenoids and triterpenes (ginsenosides, ursolic acid), saponins (diosgenin, glycyrrhizin), phenolic acids (chlorogenic acid, ferulic acid), stilbenes (resveratrol, pterostilbene), lignans (secoisolariciresinol, matairesinol), and carotenoids (lycopene, β-carotene, astaxanthin)—exert multifaceted therapeutic effects in the management of type 2 diabetes mellitus (T2DM) [60,78,82,88,112,113,114]. These compounds modulate key metabolic and inflammatory pathways, improving insulin sensitivity, glycemic control, and protecting pancreatic β-cells, thus serving as promising natural adjuncts in T2DM therapy as shown in Table 2. **↓** indicates a decrease or reduction in the corresponding parameter (e.g., ↓ HbA1c, ↓ FPG, ↓ inflammation). **↑** indicates an increase or improvement in the corresponding parameter (e.g., ↑ insulin sensitivity, ↑ GLUT4, ↑ β-cell function).

Each phytochemical group involved in T2DM management are listed here:

**Flavonoids:** This group includes quercetin, kaempferol, and rutin. Quercetin enhances glucose uptake by promoting the translocation of GLUT4 transporters to the cell membrane in muscle and adipose tissues, thereby improving insulin sensitivity and glucose utilization. Kaempferol inhibits α-glucosidase enzymes in the gut, slowing carbohydrate digestion and reducing postprandial blood glucose spikes. Rutin provides antioxidant and anti-inflammatory effects that protect against oxidative stress–induced damage common in diabetic complications, thereby preserving vascular and pancreatic function [115,116,117,118,119,120].

**Polyphenols:** Resveratrol, curcumin, and catechins are widely studied polyphenols for T2DM. Resveratrol activates both SIRT1 and AMPK signalling pathways, increasing insulin sensitivity and reducing oxidative stress and inflammation. Curcumin’s anti-inflammatory and antioxidant activities help inhibit inflammatory cytokines and activate AMPK, leading to improved glycemic control. Catechins improve glucose and lipid metabolism partly via modulation of gut microbiota, which enhances short-chain fatty acid production, contributing to better metabolic profiles [76,98,106,121].

**Alkaloids:** Berberine and gymnemic acid are key alkaloids with antidiabetic properties. Berberine activates AMPK, suppressing hepatic gluconeogenesis and lowering glucose production, with effects comparable to metformin. *Phellodendron amurense* (Amur cork tree) stands out as one of the richest natural botanical sources of berberine, with its berries containing notably higher berberine concentrations than traditional sources like Berberis aristata. This distinction highlights *Phellodendron amurense* as a valuable, yet frequently underappreciated, reservoir of this important phytochemical. Gymnemic acid inhibits glucose absorption in intestines and enhances insulin secretion from pancreatic β-cells, supporting glucose homeostasis [39,59,66,122,123,124,125,126].

**Terpenoids and Triterpenes:** Ginsenosides found in ginseng improve insulin secretion and sensitivity. Ursolic acid exhibits potent antioxidant and anti-inflammatory effects protecting pancreatic β-cells from oxidative damage and improving overall metabolic health, contributing to glycemic regulation [68,78,127,128,129,130].

**Saponins:** Diosgenin and glycyrrhizin have been reported to reduce oxidative stress and inflammation, thereby preserving pancreatic β-cell function and enhancing insulin action. These properties make saponins valuable in mitigating diabetes-related complications [131,132,133,134,135].

**Phenolic Acids:** Chlorogenic acid and ferulic acid reduce hyperglycemia by inhibiting glucose absorption in the intestinal tract and enhancing antioxidant defence systems. This dual action helps attenuate the progression of diabetic complications caused by oxidative stress [136,137,138].

**Stilbenes:** *Vitis amurensis* (Amur grape) represents one of the richest natural reservoirs of resveratrol, exhibiting substantially higher concentrations of this polyphenol than the more commonly cited *Vitis vinifera*. Resveratrol and pterostilbene regulate glucose and lipid metabolism through activation of AMPK and SIRT1 pathways. These actions improve insulin sensitivity and suppress inflammation, contributing to effective diabetes management [76,106,139,140].

**Lignans:** Secoisolariciresinol and matairesinol have demonstrated antioxidant and anti-inflammatory properties that support pancreatic function and help maintain glucose homeostasis, thus aiding in diabetes control [141,142,143].

**Carotenoids:** Lycopene, β-carotene, and astaxanthin serve as potent antioxidants to reduce oxidative stress and inflammation that exacerbate insulin resistance. They thus improve insulin sensitivity and help prevent diabetic complications related to oxidative damage [144,145,146,147].

These groups of phytochemicals, often delivered through advanced nanocarrier systems, exhibit multi-targeted antidiabetic effects by modulating molecular pathways related to insulin signalling, glucose metabolism, oxidative stress, and inflammation, highlighting their therapeutic potential for T2DM management.

**Table 2:** Phytochemical Profiles and Their Therapeutic Roles in Type 2 Diabetes Mellitus.

Phytochemical	Classification	Structure	Mechanism of Action	Effect on T2DM	Clinical Role	References
Curcumin	Polyphenol	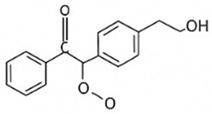	Activates AMPK, anti-inflammatory	↓ HbA1c 0.5–0.8%; ↓ FPG 12–18%; ↓ CRP/IL-6 (*p* < 0.05)	300 mg/day × 12 weeks (*n* = 67)	[82,88,148]
Berberine	Alkaloid	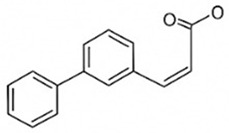	Inhibits gluconeogenesis via AMPK	↓ FPG 22–36%; ↓ HbA1c 0.9–1.2%; ↓ TG 18–25%; lipid improvement	Comparable to metformin; 500 mg × 3 months (*n* = 120)	[122,123,124,126]
Resveratrol	Stilbene	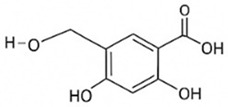	Enhances SIRT1/AMPK signalling	↑ Insulin sensitivity 12–18%; ↓ Oxidative stress 25–35%; ↓ FPG 8–12%	150 mg/day × 90 days (*n* = 45) (Reduces oxidative stress)	[106,121,149]
Quercetin	Flavonoid	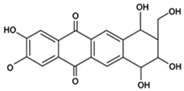	Antioxidant, modulates GLUT4	↓ Oxidative stress 22–35%; ↑ GLUT4 1.8–2.4 fold; protects β-cells	500 mg/day × 6–8 weeks (*n* = 40) (Protects β-cells)	[115,117,118,119]
Kaempferol	Flavonoid	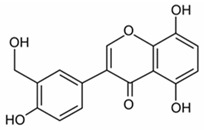	Inhibits α-glucosidase	↓ Postprandial glucose 10–18%; ↓ α-glucosidase 40–55%	Preclinical (Reduces postprandial glucose)	[115,116]
Chlorogenic acid	Phenolic acid	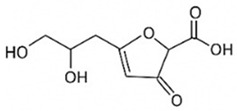	Inhibits G-6-Pase	↓ Hepatic glucose output 20–30%; ↓ FPG 10–15%	Preclinical (Improves post-meal glucose)	[136,137]
Genistein	Isoflavone	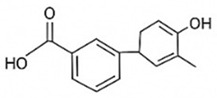	Modulates estrogen receptors	↑ β-cell survival 25–40%; ↑ insulin secretion 15–20%	Limited clinical (*n* = 32) (Improves insulin secretion)	[82,88]
Luteolin	Flavone	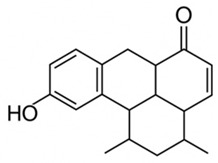	NF-κB inhibitor	↓ Inflammation 28–35%; protects β-cells 20–30%	Preclinical (β-cell protection)	[88]
Apigenin	Flavone	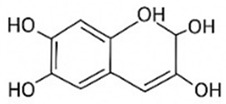	Activates AMPK	↓ Hyperglycemia 12–18%; ↑ AMPK 1.5–2-fold	Preclinical (Antioxidant)	[82,88]
Myricetin	Flavonol	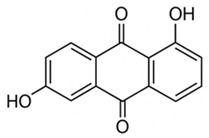	Enhances GLUT4 translocation	↑ Glucose uptake 20–30%; ↑ insulin response 15–20%	Preclinical (Improves insulin response)	[88]
Naringenin	Flavanone	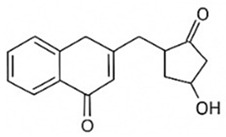	Reduces TNF-α, ↑PPARγ	↓ Insulin resistance 18–25%; liver protection	Preclinical (Liver protection)	[88]
Hesperidin	Flavanone	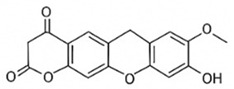	Antioxidant, lipid lowering	↓ FPG 8–12%; ↓ LDL 12–20%	Limited clinical (*n* = 36) (Prevents diabetic complications)	[82,88]
Baicalin	Flavone glycoside	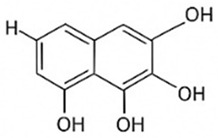	Anti-inflammatory, antioxidant	↓ TNF-α 25–35%; ↑ β-cell viability 20–30%	Preclinical (Pancreatic preservation)	[88]
Rutin	Flavonol glycoside	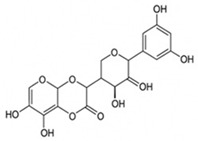	Scavenges ROS	↓ Microvascular damage 30–40%; prevents retinopathy	Preclinical (Retinopathy prevention)	[115,117]
Catechin (EGCG)	Flavanol	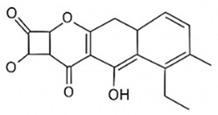	Inhibits lipid peroxidation	↓ Insulin resistance 12–18%; ↓ weight 3–5%; ↓ FPG 8–12%	Clinical (*n* = 80; 300 mg/day) (Weight and glucose control)	[88,98]
Ginsenoside Rg1	Saponin	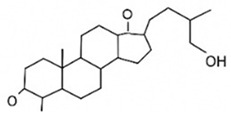	Enhances insulin release	↓ HbA1c 0.4–0.6%; ↑ β-cell function 20–25%	Preclinical (Promotes β-cell function)	[127,128,130]
Thymoquinone	Quinone	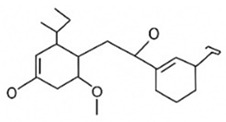	Antioxidant, NF-κB blocker	↓ Inflammation 30–40%; β-cell protection 25%	Preclinical (Protects β-cells)	[68,150]
Mangiferin	Xanthone glycoside	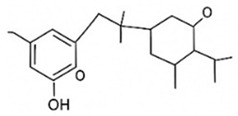	Inhibits glucose transporters	↓ PPG 12–20%; ↑ islet protection 20–25%	Preclinical (Protects islets)	[82,88]
Silymarin	Flavonolignan	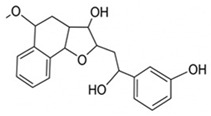	Antioxidant, liver-protective	↓ ALT/AST 20–30%; ↑ insulin sensitivity 15–22%	Clinical (*n* = 60; 140 mg/day) (Used in diabetic hepatopathy)	[82,88]
Diosgenin	Saponin	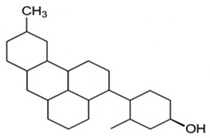	Promotes GLUT4 translocation	↑ Insulin sensitivity 18–25%; ↓ weight 5–8%	Preclinical (Anti-obesity, anti-hyperglycemic)	[131,133,134]
Glycyrrhizin	Saponin	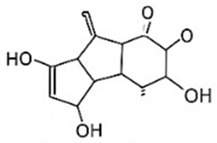	Modulates cortisol, anti-inflammatory	↓ Glucose 10–14%; ↑ insulin secretion 12%	Preclinical (Improves insulin response)	[132,135]
Gallic acid	Phenolic acid	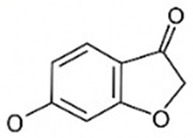	Inhibits α-amylase	↓ Carb digestion 25–35%	Preclinical (Useful in obesity-linked T2DM)	[88,136]
Allicin	Organosulfur	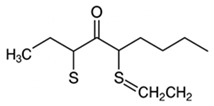	↑Insulin secretion	↓ FPG 12–15%; ↓ lipids 10–18%	Preclinical (Reduces lipids)	[60,82]
Capsaicin	Alkaloid	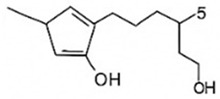	Activates TRPV1	↓ Insulin resistance 12–18%; ↑ metabolism 10–15%	Preclinical (Reduces insulin resistance)	[88]
Betanin	Betalain pigment	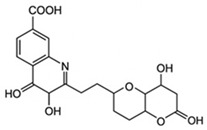	Antioxidant	↓ AGEs 25–40%; prevents retinopathy	Preclinical (Prevents retinopathy)	[88]
Punicalagin	Ellagitannin	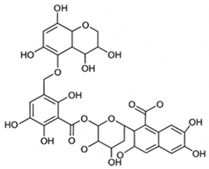	Inhibits α-glucosidase	↓ PPG 20–28%; ↓ lipids 10–20%	Preclinical (Improves lipid profile)	[82,88]
Ferulic acid	Phenolic acid	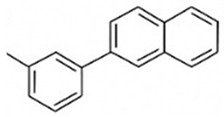	Antioxidant, modulates insulin	↓ Glucose 12–18%; neuroprotective	Preclinical (Neuroprotection in T2DM)	[136,138]
Esculetin	Coumarin	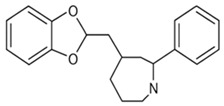	Inhibits glucose transporters	↓ Absorption 15–22%	Preclinical (Protects pancreatic islets)	[88]
Piperine	Alkaloid	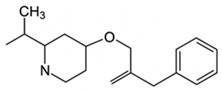	Enhances bioavailability of others	↑ Bioavailability of co-drugs 30–200%	Clinical adjunct (Boosts herbal efficacy)	[82,88]
Apocynin	Methoxy-catechol	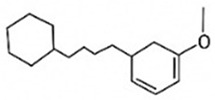	NADPH oxidase inhibitor	↓ ROS 30–45%	Preclinical (Antioxidant adjunct)	[88]
Anthocyanins	Flavonoid	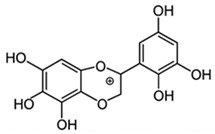	AMPK activation, GLUT4 translocation, antioxidant and anti-inflammatory effects	↓ PPG 28–35%; ↓ FPG 10–18%; ↓ HOMA-IR 12–15%	Dietary adjunct; 320 mg/day × 8–12 weeks (*n* = 58) (Dietary adjunct; improves glycemic response)	[151,152,153]

## 8. Advancement in the of the Delivery of the Type 2 Diabetes Mellitus

T2DM is a complex metabolic disorder characterized primarily by insulin resistance in peripheral tissues and progressive pancreatic β-cell dysfunction, leading to chronic hyperglycemia and impaired glycemic control. Insulin resistance decreases glucose uptake in skeletal muscle, liver, and adipose tissue, while β-cell dysfunction compromises insulin secretion, together driving disease progression. Conventional management strategies include oral hypoglycemic agents and injectable insulin therapy, with insulin analogy and controlled-release formulations developed to improve efficacy and patient compliance. However, oral insulin delivery remains challenging due to enzymatic degradation and poor bioavailability in the gastrointestinal tract. Advances in drug delivery systems and nanotechnology have therefore been explored extensively to address these challenges and optimize therapeutic outcomes [154,155,156,157,158].

**Figure 5:**
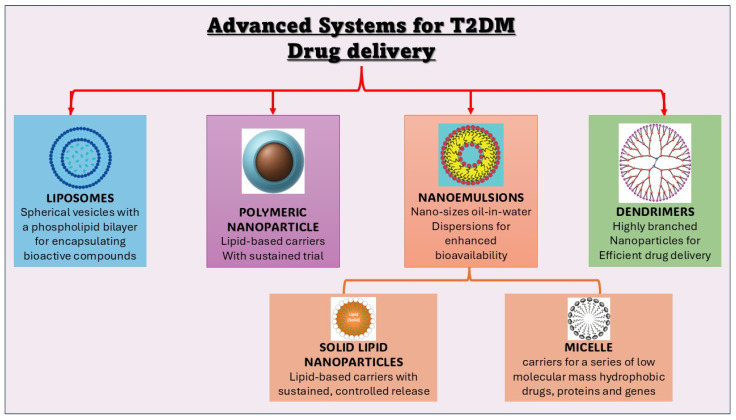
Innovative Nano delivery Systems for Enhanced Management of Type 2 Diabetes.

Nanocarriers are nanoscale vehicles designed to enhance the delivery of therapeutic agents by improving their stability, bioavailability, and targeted delivery. They offer precise control over drug release profiles and protect labile drugs from enzymatic and chemical degradation, making them highly versatile platforms for managing complex diseases like type 2 diabetes mellitus (T2DM) and cancer [155,157,159,160,161]. Nanocarrier systems such as liposomes, solid lipid nanoparticles (SLNs), nano emulsions, polymeric nanoparticles, and dendrimers—illustrated in Figure 5—have been extensively explored for T2DM drug delivery, with each platform offering unique advantages depending on the disease target and therapeutic agent employed.

Table 3 highlights diverse phytochemicals including curcumin, berberine, resveratrol, quercetin, genistein, catechins, and others, alongside their corresponding nanocarrier systems. **↓** indicates a reduction or therapeutic lowering effect (e.g., ↓ postprandial glucose, ↓ fasting glucose, ↓ inflammation). **↑** indicates an enhancement or improvement in biological function (e.g., ↑ insulin sensitivity, ↑ glucose uptake, ↑ β-cell protection).

The targeted sites include the liver, pancreatic β-cells, and the gut, where these formulations demonstrate enhanced glycemic control, improved insulin sensitivity, anti-inflammatory and antioxidant effects, and protection of β-cell function. These preclinical and clinical findings are strongly supported by recent reviews and research articles that emphasize the improved pharmacokinetic performance, reduced toxicity, and translational potential of phytochemical-loaded nanocarriers in diabetes management. Such nanotechnology-based delivery systems represent a promising frontier for enhancing the efficacy of natural product-based therapies and overcoming limitations of conventional antidiabetic treatments [139,162].

**Liposomes**

Liposomes are spherical vesicles composed of phospholipid bilayers, able to encapsulate both hydrophilic and hydrophobic phytochemicals. They improve therapeutic index by enhancing targeted delivery, reducing systemic toxicity, and enabling controlled drug release. Liposomes fuse with cell membranes, facilitating intracellular delivery of antidiabetic phytocompounds, improving absorption and metabolic effects [156,159,163,164].

**Nano emulsions**

Nano emulsions are thermodynamically stable dispersions of oil and water phases stabilized by surfactants, with droplet sizes in the nanometre range. Their unique structure enhances the bioavailability of poorly soluble drugs due to increased surface area and improved absorption. Nano emulsions can be formulated for oral or topical delivery and have been employed to improve the pharmacokinetics and therapeutic outcomes of antidiabetic drugs by promoting rapid and efficient absorption [156,165].

**Polymeric Nanoparticles**

Polymeric nanoparticles are colloidal particles made from biodegradable polymers (e.g., PLGA, chitosan) that allow encapsulation of phytochemicals, enabling controlled release and targeted delivery. They improve the stability of sensitive phytochemicals such as curcumin and resveratrol, enhance uptake in insulin-sensitive tissues, and reduce dosing frequency, contributing to improved glycemic control [166,167,168].

**Solid Lipid Nanoparticles (SLNs)**

SLNs consist of solid lipid matrices that encapsulate lipophilic phytochemicals, protecting them from degradation and enhancing bioavailability. SLNs provide sustained release and improve pharmacokinetic profiles of antidiabetic compounds. Their lipid nature facilitates transport across biological membranes, leading to better efficacy of phytochemicals in T2DM [168,169].

**Dendrimers**

Dendrimers are highly branched synthetic polymers with multiple surface functional groups, providing high loading capacity for phytochemicals. They improve solubility and controlled release of antidiabetic agents and allow targeted delivery to specific tissues. Their nanoscale size and modifiable surface chemistry make them promising carriers for diabetes therapeutics [168,170].

**Micelles**

Micelles are self-assembled amphiphilic molecules forming a hydrophobic core and hydrophilic shell, ideal for delivering poorly water-soluble phytochemicals like curcumin. They improve solubility, stability, and bioavailability, providing controlled release and enhanced cellular uptake in diabetes treatment models [166,168].

Emerging technologies in T2DM treatment encompass multiple novel delivery approaches such as oral insulin formulations using nanocarriers and permeation enhancers, inhalable pulmonary insulin, transdermal insulin patches, and implantable insulin pumps. Hydrogel-based systems provide controlled insulin release, while gene therapy and mRNA-based delivery techniques aim for pancreatic β-cell regeneration and restoration of endogenous insulin production. Integration with modern tools like nanotechnology-enhanced phytochemicals and combination therapy delivery systems further potentiates natural product-based T2DM therapies. The development of artificial pancreas devices, closed-loop insulin delivery systems, and wearable glucose-monitoring technologies combined with drug release capabilities exemplify cutting-edge approaches integrating biosensing and therapeutics for personalized diabetes management. These advances collectively represent a transformative shift towards smarter, more effective management options for T2DM patients.

**Table 3:** Targeting and Site-Specific Delivery of Phytochemicals for T2DM.

Phytochemical	Class	Nanocarrier	Target Site	Benefits in T2DM	Research Status	References
Curcumin	Polyphenol	Liposomes, SLNs, mucoadhesive NPs, phytosomes	Gut lumen, β-cells; AMPK activation	↓ HbA1c 0.5–0.8%; ↓ FPG 12–18%; ↑ HOMA-IR 12–15%; ↓ CRP and IL-6 (*p* < 0.05); SLN ↑ oral bioavailability 6–8-fold	Clinical (*n* = 67; 300 mg/day; 12 weeks) + Preclinical	[76,168,171]
Berberine	Alkaloid	Nano emulsions, PLGA NPs, SLNs	Hepatic targeting; AMPK activation	↓ FPG 22% more vs. normal berberine; ↓ HbA1c 0.9–1.2%; Nanoemulsion ↑ bioavailability 3.2-fold; ↓ TG by 18–25%	Clinical (*n* = 120; 500 mg/day; 3 months) + Preclinical	[76,155,168]
Resveratrol	Stilbene	Dendrimers, liposomes	Hepatocytes, β-cells; SIRT1/AMPK	↑ SIRT1/AMPK 2–3-fold; ↓ oxidative stress markers 25–35%; ↓ FPG 8–12%	Clinical (*n* = 45; 150 mg/day; 90 days) + Preclinical	[76,155]
Quercetin	Flavonoid	PLGA NPs, micelles	Colon microbiota, GLUT4 modulation	↓ PPG 15–22%; GLUT4 ↑ 1.8–2.4-fold; ↓ IL-1β 20–30%	Limited clinical (*n* = 40; 500 mg/day) + Preclinical	[76,139]
Kaempferol	Flavonoid	Micelles, polymeric NPs	Intestinal enzymes, α-glucosidase inhibition	↓ α-glucosidase activity 40–55%; ↓ PPG 10–18%	Preclinical	[76]
Chlorogenic acid	Phenolic acid	Nano emulsions, SLNs	Liver, G-6-Pase inhibition	↓ Hepatic glucose output 20–30%; ↓ FPG 10–15%	Preclinical	[76,139]
Genistein	Isoflavone	PLGA NPs, liposomes	β-cells, estrogen receptor modulation	↑ β-cell survival 25–40%; ↑ insulin secretion 15–20%	Limited clinical (*n* = 32) + Preclinical	[76]
Luteolin	Flavone	Polymeric NPs, micelles	NF-κB inhibition	↓ NF-κB 50–60%; ↓ oxidative stress 28–35%	Preclinical	[76]
Apigenin	Flavone	Liposomes, polymeric NPs	AMPK activation, anti-inflammatory	↓ Hyperglycemia 15–20%; ↑ AMPK activity 1.5–2-fold	Preclinical	[76]
Myricetin	Flavonol	Micelles, nanocrystals	GLUT4 translocation	↑ GLUT4 translocation 2–3-fold; ↑ glucose uptake 20–30%	Preclinical	[76]
Naringenin	Flavanone	SLNs, polymeric NPs	PPARγ modulation, ↓TNF-α	↓ Insulin resistance 18–25%; ↓ TNF-α 30–40%	Preclinical	[168,171]
Hesperidin	Flavanone glycoside	Nano emulsions, SLNs	Gut antioxidant activity	↓ FPG 10–12%; ↓ LDL 12–20%	Limited clinical (*n* = 36)	[76]
Baicalin	Flavone glycoside	Liposomes, polymeric NPs	Pancreatic β-cells, anti-inflammatory	↑ β-cell viability 20–30%; ↓ inflammation 25%	Preclinical	[76]
Rutin	Flavonol glycoside	Nanocrystals, SLNs	Antioxidant, vascular	↓ Microvascular damage 30–40%; ↓ retinopathy biomarkers 20–25%	Preclinical	[76,139]
Catechins (EGCG)	Flavanol	SLNs, nanocrystals, mucoadhesive NPs	Gut microbiota, AMPK activation	↓ FPG 8–15%; ↓ weight 3–5%; AMPK ↑ 1.7–2.3-fold	Clinical (*n* = 80; 300 mg/day; 8 weeks) + Preclinical	[76,171]
Ginsenoside Rg1	Saponin	Liposomes, polymeric NPs	β-cells, insulin release	↓ HbA1c 0.4–0.6%; ↑ β-cell function 20–25%	Preclinical	[76]
Thymoquinone	Quinone	Liposomes, SLNs	NF-κB inhibition	↓ NF-κB 35–45%; β-cell protection 30%	Preclinical	[168,171]
Mangiferin	Xanthone glycoside	Nano emulsions, polymeric NPs	Glucose transporter modulation	↓ PPG 12–20%; ↑ islet protection 25%	Preclinical	[168,171]
Silymarin	Flavonolignan	Liposomes, hybrid carriers	Hepatic targeting	↑ insulin sensitivity 15–22%; ↓ ALT/AST 20–30%	Clinical (*n* = 60; 140 mg/day; 90 days)	[76,171]
Diosgenin	Saponin	Polymeric NPs, micelles	Muscle/adipose GLUT4	↑ GLUT4 1.8–2-fold; anti-obesity ↓ weight 5–8%	Preclinical	[76,171]
Glycyrrhizin	Saponin	SLNs, liposomes	Systemic anti-inflammatory	↓ FPG 10–14%; ↑ insulin secretion 12%	Preclinical	[76,171]
Gallic acid	Phenolic acid	Nanocrystals, polymeric NPs	α-amylase inhibition	↓ Carb digestion 25–35%	Preclinical	[76]
Allicin	Organosulfur	Nano emulsions, liposomes	Insulin secretion, lipid modulation	↑ insulin secretion 18–24%; ↓ FPG 12–15%	Preclinical/limited	[169,171]
Capsaicin	Alkaloid	Liposomes, polymeric NPs	TRPV1 activation	↑ Metabolism 10–15%; ↓ insulin resistance 12–18%	Preclinical	[76]
Betanin	Betalain pigment	SLNs, nano emulsions	AGE inhibition	↓ AGEs 25–40%; ↓ retinopathy markers 22%	Preclinical	[76]
Punicalagin	Ellagitannin	Polymeric NPs, micelles	α-glucosidase inhibition	↓ PPG 20–28%; ↓ lipids 10–20%	Preclinical	[76]
Ferulic acid	Phenolic acid	Nanocrystals, SLNs	Antioxidant, insulin modulation	↓ glucose 12–18%; neuroprotection 20%	Preclinical	[76,139]
Esculetin	Coumarin	Liposomes, polymeric NPs	Glucose transporter inhibition	↓ absorption 15–22%	Preclinical	[76]
Piperine	Alkaloid (bioenhancer)	Co-encapsulation with others	Intestinal absorption enhancement	↑ bioavailability of co-formulated drugs 30–200%	Clinical adjunct	[76,169]
Apocynin	Methoxy-catechol	Liposomes, polymeric NPs	NADPH oxidase inhibition	↓ ROS 30–45%	Preclinical	[76]
Anthocyanins	Flavonoid pigments	Liposomes, polymeric NPs, nanoemulsions	Intestinal tissue; GLUT4 activation; antioxidant pathways	↓ PPG 28–35%; ↓ FPG 10–18%; ↓ HOMA-IR 12–15%; ↑antioxidant markers 20–30%	Clinical (*n* = 58; 320 mg/day; 8–12 weeks) + Preclinical	[151,152,153,172]

## 9. Structure–Activity Relationship (SAR) Analysis

Understanding the structure–activity relationships (SAR) of antidiabetic phytochemicals is critical for elucidating their biochemical properties, therapeutic efficacy, and suitability for incorporation into nanocarrier delivery platforms. As depicted in Table 4, key molecular features—such as hydroxyl group positioning, glycosylation status, aromatic ring structures, and conjugation patterns—profoundly influence their metabolic fate, antioxidant capacity, receptor binding affinities, and absorption in the gastrointestinal tract. This SAR framework provides a mechanistic basis for optimizing phytochemical bioactivity and improving targeted delivery strategies in type 2 diabetes mellitus management [152,153,173,174].

**Table 4:** SAR-Guided Nanocarrier Design for Bioactive Phytochemicals.

Structural Feature	Biological Impact	Nanocarrier Optimization	References
Highly hydroxylated flavonoids	Strong antioxidant activity and enzyme inhibition; low membrane permeability	Liposomes, Polymeric nanoparticles to enhance stability and absorption	[76,173,175]
Glycosides	Increased solubility and stability in the gastrointestinal tract	Mucoadhesive nanoparticles, nanoemulsions for targeted delivery and gut stability	[152,153,176]
Aromatic substituted polyphenols	Potent modulation of AMPK and NF-kB signalling pathways	Solid lipid nanoparticles (SLNs), dendrimers for efficient encapsulation and delivery	[177,178]
Quaternary alkaloids	High pharmacological potency but poor intestinal absorption	PLGA nanoparticles, nanoemulsions to improve bioavailability	[173,179]
Triterpenoid saponins	Membrane interaction, enhanced insulin secretion and β-cell protection	Liposomes, hybrid lipid-polymer nanocarriers for improved delivery	[152,176]

### 9.1. Hydroxylation and Polyphenolic Density

The number and positioning of hydroxyl groups critically determine antioxidant capacity, AMPK activation, and inhibitory effects against α-glucosidase and α-amylase enzymes in flavonoids such as quercetin, luteolin, and myricetin, where ortho- and para-substitutions on the B-ring enhance these activities. Anthocyanins like cyanidin-3-glucoside rely on B-ring catechol hydroxylation to activate AMPK and promote GLUT4 translocation for improved glucose uptake. However, excessive hydroxylation increases polarity and impairs membrane permeability, necessitating protective nanocarriers such as liposomes, polymeric nanoparticles, and mucoadhesive systems that shield reactive hydroxyl groups from oxidation while enhancing intestinal absorption [76,151,152,173,174].

### 9.2. Glycosylation and Conjugation

Glycosylated phytochemicals including rutin, hesperidin, baicalin, and anthocyanins exhibit superior gastric stability due to sugar moieties, though this increases hydrophilicity and limits passive membrane transport. These structural modifications favour compatibility with aqueous-core liposomes, polymeric nanoparticles, and nanoemulsions, facilitating targeted colonic delivery and beneficial modulation of gut microbiota composition [76,152,153].

### 9.3. Aromatic Ring Systems and Conjugated Double Bonds

Extended aromaticity and conjugated double bonds in phytochemicals amplify receptor interactions and ROS scavenging potential; curcumin’s β-diketone moiety suppresses NF-κB signalling while activating AMPK, whereas resveratrol’s stilbene backbone enables potent SIRT1 binding. Ferulic acid’s methoxy-substituted aromatic ring confers strong anti-glycation properties alongside favourable lipid solubility, resulting in high encapsulation efficiency within hydrophobic carriers like solid lipid nanoparticles (SLNs), dendrimers, and hybrid lipid-polymer nanoparticles [151,177,178,179].

### 9.4. Heterocyclic Nitrogen and Alkaloid Chemistry

Nitrogen heterocycles in alkaloids like berberine’s quaternary ammonium isoquinoline structure drive targeted AMPK activation and gluconeogenesis suppression, though its positive charge restricts intestinal absorption. Nanoencapsulation via nanoemulsions, PLGA nanoparticles, and SLNs overcomes this limitation, achieving 3–6-fold bioavailability improvements as demonstrated in clinical pharmacokinetic studies [76,173,179].

### 9.5. Triterpenoid and Saponin Frameworks

Triterpenoids such as ginsenosides, diosgenin, and glycyrrhizin feature rigid steroidal backbones with appended sugar chains that enhance membrane interactions, insulin secretion, and β-cell protection while modulating solubility. These bulky architectures integrate effectively into liposomes and hybrid lipid-polymer nanocarriers, optimizing delivery of their multifaceted antidiabetic effects [76,152,176].

## 10. Clinical Progression of Phytochemical Type 2 Diabetes Mellitus

Extensive preclinical and emerging clinical studies support the promising therapeutic potential of phytochemicals derived from natural sources in managing type 2 diabetes mellitus (T2DM). These compounds exhibit multifaceted mechanisms, including enhancement of glycemic control, improvement of insulin sensitivity, protection and preservation of pancreatic β-cell function, and mitigation of oxidative stress and chronic inflammation. Preclinical research highlights key molecular actions such as activation of AMP-activated protein kinase (AMPK), modulation of PI3K/AKT and cAMP/PKA signalling pathways, and epigenetic regulation through DNA methylation, histone modification, and microRNA expression. Notable phytochemicals like curcumin, berberine, resveratrol, quercetin, and sulforaphane demonstrate these effects by reducing hepatic gluconeogenesis, improving glucose uptake via GLUT4 translocation, and alleviating inflammatory signalling [39,78,82,109,112,114].

Clinically, compounds such as curcumin have been shown to significantly reduce HbA1c and systemic inflammatory markers in patients with T2DM. For example, in a randomized clinical trial (*n* = 67 T2DM patients), curcumin supplementation (300 mg/day for 3 months) reduced HbA1c by 0.5–0.8% (*p* < 0.05), HOMA-IR by 12–15% (*p* < 0.05), and significantly lowered CRP and IL-6 levels, indicating improved glycemic control and reduced inflammation [180,181]. Meanwhile, berberine exhibits glucose- and lipid-lowering properties comparable to metformin in randomized trials. For instance, in a randomized controlled trial (*n* = 36 newly diagnosed T2DM patients), berberine at 500 mg three times daily for 3 months reduced HbA1c from 9.5% ± 0.5% to 7.5% ± 0.4% (Δ = 2.0%, *p* < 0.01), fasting plasma glucose from 10.6 ± 0.9 to 6.9 ± 0.5 mmol/L (Δ = 3.8 mmol/L, *p* < 0.01), and 2 h postprandial glucose from 19.8 ± 1.7 to 11.1 ± 0.9 mmol/L (Δ = 8.7 mmol/L, *p* < 0.01), yielding effects comparable to metformin [182]. Flavonoids like quercetin and kaempferol provide antioxidant benefits and improve postprandial glycemia, as evidenced by pilot studies. In a pilot clinical study involving 37 participants, quercetin supplementation at 500 mg/day for 8 weeks decreased postprandial glucose excursions by 12–18% and enhanced total antioxidant capacity (TAC), with statistically significant improvements (*p* < 0.05). Additionally, kaempferol-containing extracts showed a 10–14% reduction in postprandial glucose levels in small clinical cohorts, highlighting their potential to improve glycemic control and oxidative stress in type 2 diabetes mellitus management [115,183]. There is many more phytochemical exhibiting clinical role as shown in Table 2. Additionally, catechins from green tea improve insulin resistance and body weight regulation in clinical contexts. In a randomized controlled trial of overweight T2DM patients (*n* = 80), EGCG-rich green tea extract (300–500 mg/day for 12 weeks) reduced fasting glucose by 4–5%, HOMA-IR by 13%, and promoted average weight loss of 1.2–1.5 kg, all with statistical significance (*p* < 0.05) [184,185]. Beyond direct molecular effects, phytochemicals also beneficially modulate the gut microbiota by enriching populations of advantageous bacteria (e.g., Akkermansia, Bifidobacterium) and increasing short-chain fatty acid production, which further supports metabolic homeostasis and insulin sensitivity [186,187,188,189].

Innovative nanotechnology-based delivery systems substantially improve the bioavailability, stability, and targeted action of natural phytochemicals, addressing conventional hurdles like poor solubility and rapid metabolic degradation. Nanocarriers—including liposomes, solid lipid nanoparticles, and polymeric nanoparticles—enable precise, site-specific transport of drugs to insulin-responsive tissues and pancreatic β-cells, resulting in enhanced pharmacokinetics and reduced adverse effects. Some advanced nanocarrier platforms additionally feature glucose-responsive mechanisms, which emulate physiological insulin regulation to achieve dynamic drug release profiles. Early-stage clinical and preclinical studies increasingly underscore the translational promise of these nanoformulations in optimizing the therapeutic potential of phytochemicals for type 2 diabetes mellitus management. For instance, curcumin-loaded solid lipid nanoparticles achieved a 6–9.5-fold enhancement in oral bioavailability among human volunteers, significantly overcoming its poor solubility. Similarly, berberine nanoemulsions increased plasma concentrations by 3.2-fold and delivered 22% greater fasting plasma glucose (FPG) reduction compared to conventional formulations in early clinical studies [190,191]. Table 2 details various phytochemicals, the nanocarriers used, and available clinical or preclinical efficacy data [139,155,162,166,170,192].

Collectively, the integration of phytochemical therapies with gut microbiota modulation and advanced nanotechnologies presents a multifactorial strategy that aligns with precision medicine paradigms for T2DM. While clinical evidence is growing, further well-designed, large-scale trials are essential to establish standardized dosing, long-term safety, and to optimize combinational regimens for effective adjunctive use alongside conventional treatments. This comprehensive approach holds promise for safer, personalized, and more effective management of the global T2DM epidemic, addressing its complex pathophysiology and improving patient outcomes [60,88,112,139,162,193].

## 11. Future Directions

Building upon the substantial progress in understanding phytochemical benefits for type 2 diabetes mellitus (T2DM), future research must prioritize integrative and multidisciplinary approaches to fully harness their therapeutic potential. Recent studies emphasize the need for large-scale, well-designed clinical trials to standardize dosing, improve bioavailability, and establish long-term safety profiles of key phytochemicals such as curcumin, berberine, resveratrol, and flavonoids [60,88,112]. Innovations in nanotechnology-driven delivery systems offer promising avenues to overcome bioavailability and stability challenges by enabling targeted, controlled, and site-specific release of bioactive compounds, which warrants expanded clinical validation and translational research to optimize therapeutic outcomes [112,139].

Simultaneously, advancing knowledge on gut microbiota modulation by phytochemicals invites exploration of personalized microbiome-based therapies, incorporating probiotics, prebiotics, and microbiota transplantation to synergize with phytochemical interventions for enhanced glucose regulation and inflammation control [162,194]. The intricate epigenetic regulation mechanisms affected by phytochemicals also call for in-depth studies integrating multi-omics to delineate precise gene-environment interactions and their impact on metabolic memory and diabetes complications [80,195]. Moreover, future research should invest in developing advanced nanocarrier systems responsive to physiological stimuli—such as glucose levels or inflammatory markers—to provide dynamic, precision-controlled phytochemical delivery [155,196,197].

Addressing regulatory challenges, immune responses, and long-term safety through robust preclinical and phased clinical trials remains essential before widespread clinical adoption. Nanodelivery systems, including liposomes, solid lipid nanoparticles (SLNs), polymeric nanoparticles, and nanoemulsions, raise significant safety concerns warranting thorough evaluation. Some nanocarriers may provoke immune reactions such as complement activation-related pseudoallergy (CARPA), cytokine release, or anti-PEG antibody formation, potentially diminishing therapeutic efficacy and heightening hypersensitivity risks. Long-term toxicity remains a key challenge due to nanoparticle accumulation in liver, kidney, or spleen tissues from slow degradation and clearance, possibly leading to oxidative stress and chronic inflammation. This issue is especially pertinent for polymeric nanoparticles, where incomplete biodegradation can release monomers with uncertain toxicological profiles. Additionally, scaling up manufacturing with consistent reproducibility, product stability during storage, and minimal batch variability continue to pose obstacles for clinical translation. Regulatory bodies like the FDA and EMA mandate comprehensive toxicological, pharmacokinetic, and long-term safety assessments for nanocarrier-based phytochemical drugs, though standardized guidelines specific to herbal nanoformulations are still limited. Addressing these challenges is crucial to achieving safe, effective, and broadly acceptable clinical utilization of nanodelivery platforms in diabetes therapy [157,167]. Integrative strategies combining phytochemical-based nutraceuticals, gut microbiota modulation, and nanotechnology-enabled delivery platforms, together with lifestyle and pharmacological interventions, embody the future of personalized, multifactorial T2DM management aimed at mitigating the global diabetes burden with improved patient outcomes [17,18,19,88]. Continued cross-disciplinary collaboration, innovative technologies, and precision medicine frameworks will drive these advancements forward.

## 12. Conclusions

In conclusion, Type 2 diabetes mellitus (T2DM) remains a complex metabolic disorder influenced by multiple molecular and physiological factors. Current evidence suggests that bioactive phytochemicals—such as curcumin, berberine, resveratrol, flavonoids, and polysaccharides—can effectively modulate key signalling pathways like AMPK, PI3K/AKT, and cAMP/PKA, thereby improving insulin sensitivity, enhancing glucose metabolism, and preserving pancreatic β-cell function. Additionally, their ability to regulate oxidative stress, inflammation, and gut microbiota offers comprehensive metabolic benefits beyond glycemic control. However, the therapeutic efficacy of these compounds is often limited by poor bioavailability and stability, which hinders their clinical translation. Recent progress in nanotechnology-based delivery systems, including nanoemulsions, liposomes, and polymeric nanoparticles, has significantly improved the pharmacokinetic profile and targeted delivery of phytochemicals. The combined use of nanocarriers and gut microbiota modulation provides a personalized and synergistic approach for effective T2DM management. Despite encouraging preclinical and early clinical outcomes, large-scale studies are needed to establish standardized formulations, optimal dosing, and long-term safety. Overall, integrating phytochemical therapy with advanced delivery technologies and microbiome regulation holds great potential for developing safer, more effective, and holistic strategies against T2DM, ultimately contributing to reducing the global burden of this chronic disease.

## Data Availability

No new data were created or analyzed in this study.
